# Delayed Adult Gastric Perforation Following Insertion of a Feeding Nasogastric Tube

**DOI:** 10.7759/cureus.19411

**Published:** 2021-11-09

**Authors:** Mohamed Albendary, Ali Yasen Y Mohamedahmed, Anil George

**Affiliations:** 1 Department of General Surgery, Sandwell and West Birmingham National Health Services (NHS) Trust, Birmingham, GBR

**Keywords:** delayed perforation, feeding nasogastric tube, nasogastric tube complications, adult, gastric perforation

## Abstract

Although complications of a nasogastric tube (NGT) are identified and managed in daily clinical practice, gastric perforation following NGT insertion is a serious and rarely reported condition in adults. We present a case of a 71-year-old male who was brought to the hospital after having a cardiac arrest. Following stabilisation and receiving an emergency percutaneous coronary intervention (PCI), he was admitted to the intensive care unit (ICU), where he required NGT for feeding purposes. A few days later, abdominal distension was noted, and chest imaging was requested mainly for worsening respiratory parameters. A computed tomography (CT) scan confirmed gastric perforation and a misplaced NGT. Being a high-risk patient and in the absence of peritonism and frank sepsis, conservative management was adopted and included proton pump inhibitors (PPI), total parenteral nutrition (TPN), stomach aspiration via a Ryle tube and consideration of imaging-guided drainage. No risk factor for gastric perforation was identified in this presented case. The stable course of follow-up suggested sealed perforation; however, he died due to an extensive intracardiac thrombus. Though this incidence did not contribute directly to the patient’s death, it definitely added to the overall morbidity and negatively influenced the management of the other medical conditions.

For complement, we also report a review of the ten similar cases in the literature, highlighting the associated risk factors, relevant clinical challenges, lines of management executed. The main aim of this case report is to enhance doctors' awareness of this serious complication, especially in patients with risk factors, and its diagnostic dilemmas. Early recognition and prompt intervention are recommended for a better outcome.

## Introduction

Nasogastric tube (NGT) insertion is a common procedure performed for several indications including gastric decompression (large bore, 12-16 French) and enteral feeding (small bore, 10 French). NGT placement is considered a relatively safe procedure with a low incidence of complications [1]. Perforations of the nasopharynx or alimentary tract along the route of insertion have been reported with the majority being subsequent to the use of a large-bore NGT, yet such perforations remain rare [2,3]. Although NGT-related gastric perforations have been reported frequently in neonates and infants [4], they are uncommon in adults. We report a case of a delayed gastric perforation in an adult following the insertion of a fine bore NGT. For complement, we present a review of the cases reported in the literature highlighting risk factors, related clinical challenges and lines of management executed.

## Case presentation

**Figure 1:**
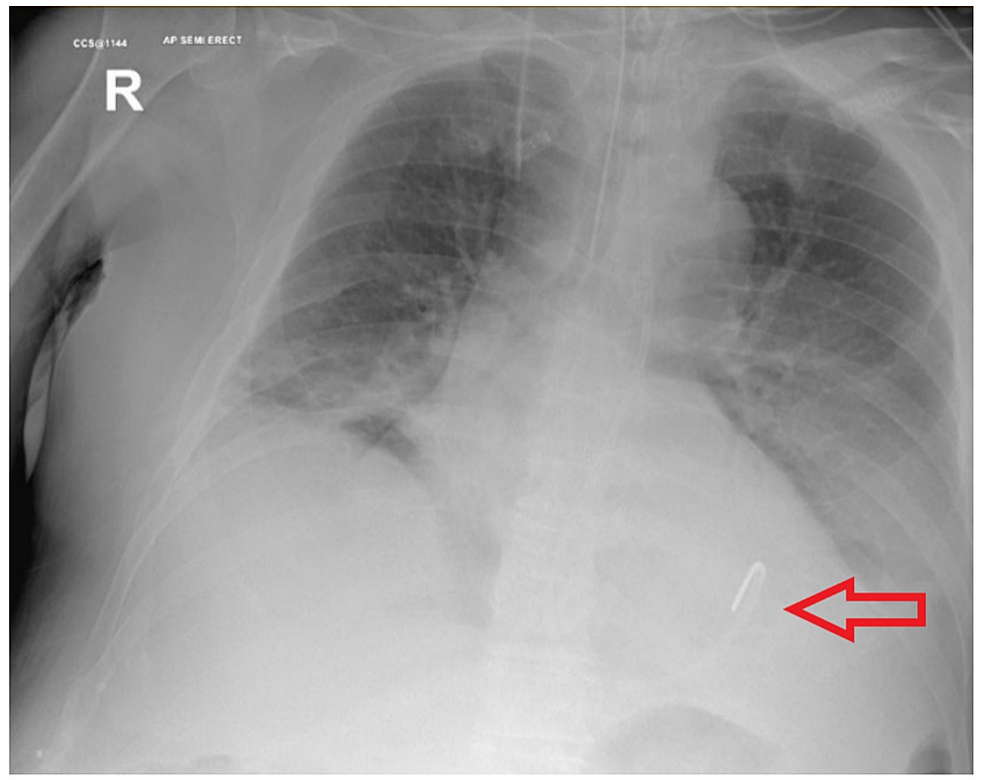
Post-placement x-ray confirming a satisfactory position.

**Figure 2:**
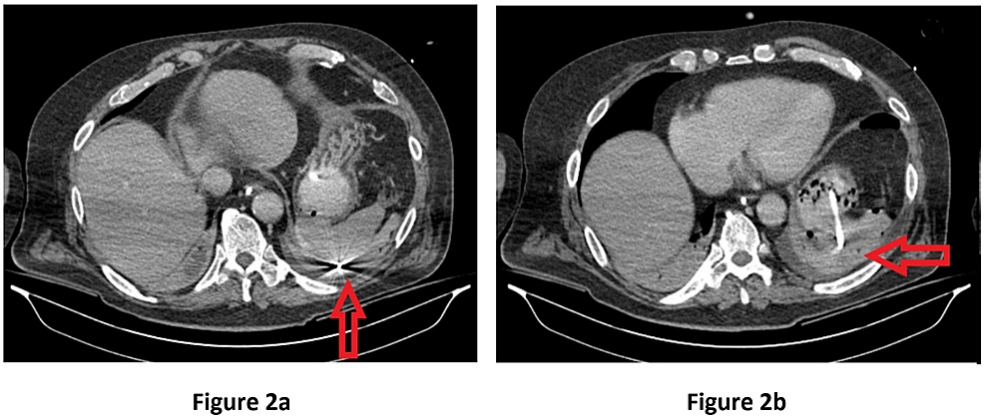
CT imaging showing a misplaced tube behind the spleen (2a) and a perisplenic collection (2b).

A 71-year-old male ventilated patient in the intensive care unit (ICU), following a cardiac arrest that required an emergency percutaneous coronary intervention (PCI), had a fine bore (10 French) NGT inserted for enteral feeding. The procedure was uneventful and followed by routine safety checks. Post-procedure x-rays confirmed apparently correct placement and feeding was subsequently started (Figure 1). It was noted that he had four different tubes inserted in his first week of ICU stay. Five days following the last NGT insertion, the patient developed abdominal distention and worsening respiratory parameters. A contrast computed tomography (CT) scan of the chest confirmed a gastric perforation with the tip of the NGT lying behind the spleen, as well as a perisplenic collection with localised locules of gas (Figures 2a, 2b).

As the patient’s condition remained stable with no peritonism on clinical examination, in addition to being a high-risk candidate for surgery due to the ongoing medical issues, conservative management was adopted. The management included proton pump inhibitor (PPI) infusion, total parenteral nutrition (TPN), regular gastric aspiration through a wide bore Ryle tube, and consideration of imaging-guided drainage. Eventually, the stability of his physiological parameters and clinical status suggested a sealed or contained perforation. The patient died a week later due to a massive intra-cardiac thrombus.

## Discussion

Though NGT placement is a common procedure with perceived low morbidity and mortality, the National Patient Safety Agency (NPSA) in the UK reported 79 harm incidents and 21 cases of mortality related to NGT over a five-year period (2005-2010) [5]. Discomfort, acid reflux, aspiration, and tube-related issues as dislodgement, displacement, and blockage are common and less morbid complications; however, luminal perforations are serious events and can be fatal [6]. Adults are more at risk of perforations of the oesophagus and pharyngo-oesophageal region following NGT placement compared to infants who are more liable to gastric perforations [7]. In the scope of neonatal cases, frequently reported in preterm and low birth weight infants, NGT insertion is the commonest cause for iatrogenic gastric perforations [4]. The main diagnostic tool is an x-ray with or without a contrast agent injected through the NGT. The reported cases were mainly treated surgically, which is rational as infants develop peritonitis abruptly, compared to adults [8-10].

Adult gastric perforations following NGT placement remain infrequent, with only 10 reported cases in the literature (Table 1) [7,11-16]. Most of these patients had one or more risk factors, which could potentially include severe gastritis, peptic ulcer disease, gastric cancer, previous gastric surgery, and prolonged steroids or non-steroidal anti-inflammatory drugs (NSAIDS) use (Table 1). Additionally, this complication was attributed to a pathological gastric wall involved in a hiatus hernia [11] and fibromuscular dysplasia [7]; however, it can happen without any identifiable precipitating factor [16]. As for our patient, he not only developed a gastric perforation secondary to a fine bore NGT but also had no risk factor identified.

**Table 1:** Reported cases of gastric perforations due to NGT in adults NSAIDS: Non-Steroidal Anti-Inflammatory Drugs, GOJ: Gastro-Oesophageal Junction, GORD: Gastro-Oesophageal Reflux Disease, OGD: Oesophago-Gastro-Duodenoscopy, NGT: Nasogastric Tube

Author	Patient age/gender	Perforation site	Potential risk factors	Management/outcome
Ghahremani, 1980 [11]	74, Female	Anterior wall/ lesser sac	NSAIDS use, Hiatus hernia	Thoracotomy repair/Recovered
	47, Male	Anastomotic line	Previous gastrectomy	Laparotomy repair/Recovered
	63, Female	Greater curvature	Not identified	Discovered on follow up/Recovered
	61, Male	Anterior wall	Gastric cancer	Not fit/Died
Lowham, 1996 [12]	Average age of 57	Anterior GOJ	GORD, Oesophagitis	Laparoscopic repair/Recovered
Lee, 2007 [13]	63, Male	Fundus	Not identified	Endoscopic clipping/Died from Pneumonia
Daliya, 2012 [7]	32, Male	Not mentioned	Fibromuscular dysplasia	Laparotomy repair/Recovered
Guttmann, 2011 [14]	79, Male	Not mentioned	Stylet/guidewire used on insertion	Not fit for surgery/Died
Janicki, 2015 [15]	78, Male	Greater curvature	Peptic ulcer	2 laparotomies + OGD/Died from pneumonia
Aeschbacher, 2018 [16]	71, Male	Not mentioned	Not identified	2 endoscopic clipping + Image-guided drainage/Recovered

Undoubtedly, it remains challenging to explain how an NGT can perforate a healthy and well-vascularised stomach of an adult. One theory is pressure ulceration and increased rigidity of NGT left in situ for a long time. A retained tube for more than five days was reported to show colour changes and become more rigid, perhaps due to the effect of the acidic gastric secretions [11]. Another potential explanation could be the continuous irritation caused when the tip of the tube impinges the layers of the alimentary tract. This is adapted from the mechanism by which ventriculoperitoneal shunts can perforate the gastrointestinal tract [17]. We could not satisfactorily explain the exact sequence of events that may have caused a 10-French feeding NGT to cause a delayed gastric perforation. Although pH testing of the aspirate and post-procedural x-ray are well-established methods used to confirm the safety and position of the NGT, these checks have some limitations. The gastric pH can be altered by medications as PPI, and it can be infeasible to aspirate the stomach through fine-bore tubes. Also, an x-ray is liable for misinterpretation and can delay using the NGT [18]. During the period from 2005 to 2010, the NPSA reported 45 incidents and 12 deaths due to x-ray misinterpretation, in addition to 16 incidents and three deaths after either obtaining an acidic pH or ignoring the obtained abnormal pH [5].

Interestingly, gastric perforations occasionally can be masked clinically leading to delayed recognition and higher morbidity as noted in gastric perforations after endoscopy [19]. Another clinical challenge is faced upon assessing sedated, paralysed, and ventilated patients, as in our reported case. Also, gastric perforations can mimic other clinically similar conditions in specific situations, as reported when masquerading as a delayed gastric sleeve leak [20]. Certainly, early diagnosis is the key to lessening morbidity and mortality. As x-ray can be misleading, a contrast CT imaging, preferably with a water-soluble contrast agent given through the tube, is a valuable non-invasive tool to confirm a diagnosis. In our presented case, there was no clinical suspicion of perforation; however, the worsening respiratory parameters triggered the urge for imaging.

The management of such conditions remains inconsistent and the ongoing medical issues and fitness for surgery can dramatically guide the treatment. The treatment consists mainly of operative repair, yet less invasive approaches as endoscopic clipping of the perforation, imaging-guided drainage of collections, and conservative management could be attempted in selected patients. The surgical intervention should be performed instantly if indicated based on the patient's condition.

## Conclusions

We report a case of delayed gastric perforation following a fine bore NGT placement. Overall, NGT insertion is a safe procedure with low morbidity and manageable adverse events; however, alimentary tract perforations can be serious. Measuring the pH of the aspirate and performing x-rays to check NGT positioning is useful, yet their limitations and liability to misinterpretation should be well recognised. Although NGT-related gastric perforations remain extremely rare, doctors need to be aware of this complication, particularly in patients with risk factors and those who may be paralysed and ventilated. Early recognition and prompt surgical intervention, if indicated, result in a better outcome.
